# Differential Epigenetic Compatibility of *qnr* Antibiotic Resistance Determinants with the Chromosome of *Escherichia coli*

**DOI:** 10.1371/journal.pone.0035149

**Published:** 2012-05-04

**Authors:** María B. Sánchez, José L. Martínez

**Affiliations:** Departamento de Biotecnología Microbiana, Centro Nacional de Biotecnología, CSIC, Cantoblanco, Madrid, Spain; Vrije Universiteit Brussel, Belgium

## Abstract

Environmental bacteria harbor a plethora of genes that, upon their horizontal transfer to new hosts, may confer resistance to antibiotics, although the number of such determinants actually acquired by pathogenic bacteria is very low. The founder effect, fitness costs and ecological connectivity all influence the chances of resistance transfer being successful. We examined the importance of these bottlenecks using the family of quinolone resistance determinants Qnr. The results indicate the epigenetic compatibility of a determinant with the host genome to be of great importance in the acquisition and spread of resistance. A plasmid carrying the widely distributed QnrA determinant was stable in *Escherichia coli*, whereas the SmQnr determinant was unstable despite both proteins having very similar tertiary structures. This indicates that the fitness costs associated with the acquisition of antibiotic resistance may not derive from a non-specific metabolic burden, but from the acquired gene causing specific changes in bacterial metabolic and regulatory networks. The observed stabilization of the plasmid encoding SmQnr by chromosomal mutations, including a mutant lacking the global regulator H-NS, reinforces this idea. Since quinolones are synthetic antibiotics, and since the origin of QnrA is the environmental bacterium *Shewanella algae*, the role of QnrA in this organism is unlikely to be that of conferring resistance. Its evolution toward this may have occurred through mutations or because of an environmental change (exaptation). The present results indicate that the chromosomally encoded Qnr determinants of *S. algae* can confer quinolone resistance upon their transfer to *E. coli* without the need of any further mutation. These results suggest that exaptation is important in the evolution of antibiotic resistance.

## Introduction

The study of the emergence and spread of antibiotic resistance is clearly important with respect to human health, and offers one of the few possibilities of following evolution in real time. Learning the mechanisms involved in the recruitment and spread of resistance genes to human bacterial pathogens is therefore important from an evolutionary perspective. It has been indicated that the resistance genes that have been acquired by pathogens via horizontal gene transfer (HGT) likely originated in antibiotic-producing bacteria, in which they played a self-protection role [1], [2], [3], [4]. Accordingly to that view, their function – to provide resistance to antibiotics – is therefore the same in both the original and new host. However, it is difficult to imagine that the same holds true for genes conferring resistance to synthetic antibiotics such as quinolones [5]. The quinolones are a family of widely used synthetic antimicrobial agents that target bacterial topoisomerases (DNA gyrase and topoisomerase IV) and, as a consequence, inhibit DNA replication and transcription. Given their synthetic origin, it was supposed that no quinolone resistance genes would exist, the only cause of resistance to these drugs arising through mutations in the genes encoding their targets. However, the study of multidrug (MDR) efflux pumps, which are chromosomally-encoded in all microorganisms [6], has shown that quinolones are among the most common substrates of these resistance elements, indicating that microorganisms possess genes involved in quinolone resistance despite the synthetic origin of these drugs [7]. Once this became known, it was predicted that plasmid-encoded quinolone resistance was possible [8], [9], and indeed, a plasmid-encoded quinolone resistance element (dubbed Qnr) was eventually found in a transferable plasmid in *Klebsiella pneumoniae* [10]. Since then, *qnr* genes have been described in plasmids from *Enterobacteriaceae*, and other plasmid-encoded quinolone resistance genes such as *aac(6′)-Ib-cr* [11] *qepA* and *oqxAB* [12], [13] have been found in bacterial pathogens.

Since there are no quinolone producers in nature, *qnr* genes must have originated in non-producer organisms. Indeed, the origin of the widely distributed, plasmid-encoded *qnr* determinant, *qnrA*, has been tracked to the non-antibiotic-producer bacterium *Shewanella algae* [14], and *qnr* genes have been found in the chromosomes of different bacteria, most of which occupy aquatic habitats [15]. The fact that chromosomally-encoded *qnr* genes are not flanked by elements involved in gene mobility, that they show strong sequence conservation, and that synteny is maintained across the strains of a given species, indicates that they have not been recently acquired by these species; rather they are the origin of the *qnr* genes involved in quinolone resistance in bacterial pathogens. This raises important evolutionary questions. Quinolones are synthetic compounds that were not present in natural ecosystems at the time of the evolution of chromosomally-encoded *qnr* determinants, so the original function of the latter is not to provide resistance to quinolones. Which evolutionary process, then, allowed the conversion of a gene not involved in resistance into an element that provides resistance against a synthetic compound not present in natural ecosystems? [16] In other words, has the exposure of microorganisms to human-produced quinolones influenced the evolution of these otherwise housekeeping genes? Further, given that there are several *qnr* genes that can potentially be transferred to a heterologous host [15], why are only a few of them currently present in the plasmids found in human pathogens?

To explain the above, the existence of pre-resistance genes able to recognize chemical moieties similar to antibiotics has been suggested. These pre-resistance genes would not confer resistance by themselves, but might easily evolve under antibiotic selective pressure to finally result in a *bona fide* antibiotic resistance determinant [17], [18]. Alternatively, a gene with a functional role different to resistance in its original host, might confer resistance without any modification [19]. This type of evolution, in which the function of a given determinant alters as a consequence of changes in the environment (i.e., not because of changes in the sequence of the gene encoding it) has been termed exaptation [20], [21]. In the case of antibiotic resistance, exaptation is linked to the metabolic and regulatory de-contextualization suffered by these genes when jumping from the original host chromosome to that of a novel host with a different metabolism and regulatory networks (for a review of this concept see [19]).

Despite the large number of resistance genes present in environmental bacteria, which could serve as donors of resistance [18], [22], [23], the actual variability of resistance genes acquired by HGT in pathogenic bacteria is very small. This might be explained by the founder effect, i.e., once a resistance gene is introduced into a plasmid capable of spreading among the most important clones of a bacterial pathogen, its displacement by a new determinant conferring resistance to the same antibiotic would be difficult (for a discussion on this concept see [24]). Alternatively, fitness costs might impede the dissemination of a given gene [25], [26]. However, it might be predicted that the fitness costs of antibiotic resistance determinants belonging to the same structural family, and thus with the same mechanism of action, would be very similar unless their levels of expression differ [27].

To gain more insight into the basic mechanisms driving the evolution of antibiotic resistance, we focused on *qnr* genes as models. Over the last few decades the *qnrA* gene has spread widely among populations of *Enterobacteriaceae*, conferring protection against quinolones upon them. *Shewanella algae*, the origin of *qnrA*, rarely produces human infections, and is therefore not significantly exposed to quinolones in its natural habitat. We also studied the Sm*qnr* gene, naturally present in the chromosome of the opportunistic pathogen *Stenotrophomonas maltophilia* [28], which confers low-level resistance to quinolones in heterologous hosts [15], [29]. Since *S. maltophilia* is more commonly in contact with human-linked microbiota than *S. algae*, the possibility of it being a donor of *qnr* genes to *Enterobacteriaceae* is potentially greater [30]. However, while *qnrA* is a widespread gene, no transfer of Sm*qnr* has ever been documented.

To address the evolutionary mechanisms that affect the spread of *qnr* genes, we compared the stability of different *qnr* genes upon their expression in a heterologous host, and performed experimental evolution assays [31], [32] to assess the associated fitness costs and to track potential compensatory mutations. Surprisingly, the fitness costs associated with one or another Qnr determinant were very different in the same host, despite their belonging to the same structural family. These results indicate that, at least for the studied determinants, fitness costs are not necessarily a general burden linked to the energy required to keep a new mobile genetic element in a cell (and thus associated with the acquisition of a genetic platform), but might be specific for the type of resistance determinant encoded into the element (and thus to the interactions of a specific mechanism with the cellular regulatory and metabolic networks). This might provide an explanation for the stability and further spread of specific resistance genes to be considered alongside the founder effect above discussed. We also found that chromosomally-encoded *qnr* determinants belonging to the core genome of bacterial species are not pre-resistance genes that require further evolution to become resistance determinants. Rather, the main evolutionary process involved in their change of function is exaptation.

## Results

### The *qnr* Genes Present in the Chromosome of *S. algae* Confer Resistance to Quinolones

It has been suggested that antibiotic resistance can be achieved as the consequence of the evolution of pre-resistance genes, chromosomally encoded in non-producer organisms, towards resistance as the consequence of the selection of specific mutants that allow the interaction of the pre-resistance determinant with the antibiotic [17]. The first described plasmid-encoded *qnr* gene (*qnrA1*) [10] differs from those encoded in the chromosomes of *S. algae* isolates [14] by 3 or 4 amino acids. It is therefore possible that *qnrA1* evolved from a pre-resistance gene with a different role in its original host, i.e., not that of conferring resistance. To address this possibility, *qnrA1* from plasmid pMG252, *qnrA3* from *S. algae* KB-1 and *qnrA4* from *S. algae* KB-2 were cloned in the vector pGEM-T, generating the plasmids pBS18, pBS19 and pBS20 respectively. These were used to transform the *Escherichia coli* strain MC4100. As shown in Table 1, the expression of the chromosomally-encoded *qnrA* genes from *S. algae* reduced the susceptibility of the recipient *E. coli* to quinolones. This indicates that those elements are not pre-resistance determinants that must evolve through adaptive genetic changes in order to confer resistance, but rather that exaptation is an important evolutionary event in their change of function. QnrA1 was the most efficient resistance determinant among the three tested in terms of increasing MICs (Table 1). This might be explained either by its post-exaptation evolution through mutation, or, among the different alleles present in the chromosomes of different *S. algae* isolates, by its being the one that confers the strongest resistance upon heterologous hosts, and thus that which provides the greatest gain in fitness under selective pressure from quinolones.

**Table 1:** Effect of different *qnr* determinants on the susceptibility to quinolones of wild-type and *hns*-deficient *E. coli* strains.

Strain	Plasmid/*qnr*gene	MIC (mg/liter)				
MC4100 (wild-type)		MOX	OFX	CIP	LVX	GAT
	pGEM-T/None	0.032	0.032	0.006	0.016	0.012
	pBS3.25/Sm*qnr*	0.047	0.047	0.008	0.016	0.023
	pBS18/*qnrA1*	0.38	0.5	0.25	0.38	0.25
	pBS19/*qnrA3*	0.25	0.38	0.094	0.125	0.19
	pBS20/*qnrA4*	0.25	0.25	0.094	0.125	0.19
JMG100 (*hns* mutant)						
	pGEM-T/None	0.032	0.047	0.008	0.016	0.012
	pBS3.25/Sm*qnr*	0.094	0.094	0.016	0.032	0.047
	pBS18/*qnrA1*	0.38	0.5	0.19	0.38	0.25
	pBS19/*qnrA3*	0.25	0.38	0.094	0.125	0.125
	pBS20/*qnrA4*	0.25	0.25	0.125	0.125	0.125

MOX, moxifloxacin; OFX, ofloxacin; CIP, ciprofloxacin; LVX, levofloxacin; GAT, gatifloxacin.

### Stability of *qnr* Genes upon Expression in an Heterologous Host

**Figure 1:**
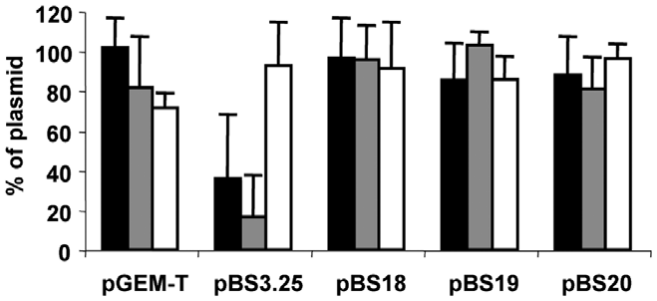
Differential fitness costs of different *qnr* determinants. Plasmid loss was measured in the *acrAB* defective *E. coli* KZM120 strain (black bars), in the wild-type strain *E. coli* MC4100 (gray bars) and in the *hns* defective *E. coli* JMG100 strain (white bars). pGEM-T, empty vector; pBS3.25, plasmid with Sm*qnr*; pBS18, plasmid with *qnrA1*; pBS19, plasmid with *qnrA3*; pBS20 plasmid with *qnrA4*. Bars show the percentage of cells containing the plasmids. As shown, the *qnrA*-containing plasmid was stable in all strains, whereas the Sm*qnr*-containing plasmid was rapidly lost, indicating that Sm*qnr* incurs a high fitness cost not observed with *qnrA*. This cost is not observed in the *hns* deficient strain, in which the Sm*qnr*-containing plasmid is maintained.

To test whether the prevalence of *qnrA* in the plasmids of bacterial pathogens might be the consequence of a founder effect or of it being associated with smaller fitness costs than other *qnr* determinants, we measured the stability in *E. coli* MC4100 of plasmids containing the different *qnrA* alleles, as well that of the plasmid pBS3.25, containing Sm*qnr*, which is chromosomally-encoded in *S. maltophilia* [15]. Single colonies of each strain were grown in LB medium with carbenicillin (a selector of the TEM1 ß-lactamase gene present in the pGEM-T backbone) overnight at 37°C, and the percentage of cells containing the plasmid determined as described in Methods. The plasmid harboring Sm*qnr* was lost in nearly 80% of the population, indicating that the acquisition of Sm*qnr* incurs a high immediate fitness cost upon *E. coli* (Figure 1). Plasmid loss can be achieved in the presence of carbenicillin since the ß-lactamase encoded by the plasmid destroys the antibiotic; thus, the selective pressure is removed a few hours after its introduction [33]. Since the efflux pump AcrAB-TolC is involved in quinolone resistance in *E. coli* [34], the same study was performed in the strain *E. coli* KZM120, which lacks *acrAB* [34], in order to avoid any problems of interference between the two types of quinolone resistance mechanism that might compromise bacterial fitness. The plasmid containing Sm*qnr* was lost in this strain as well (Figure 1).

Unlike that seen for Sm*qnr*, no plasmids encoding *qnrA* alleles were lost by either of the *E. coli* strains (Figure 1). This shows that the acquisition of *qnrA* does not impose large fitness costs on *E. coli*, and that, at least for the tested genes, the effect on bacterial fitness of different antibiotic resistance elements belonging to the same structural and functional family can be very different. The corollary of this reasoning is that resistance genes might not be replaced even if they encode proteins belonging to the same family and confer a similar phenotype - the associated fitness costs might be different for each gene. These results indicate that allelic fitness costs should be considered together with the founder effect when explaining the introduction and spread of a given resistance determinant in a population of bacterial pathogens.

The plasmids containing the different *qnr* genes used in this work have the same size and structural backbone, indicating that the observed differences in fitness costs are the consequence of a specific effect of the resistance gene and not of a non-specific burden on bacterial metabolism. Since Qnr proteins bind bacterial topoisomerases, it is possible that they alter DNA supercoiling and consequently modify global transcription and challenge bacterial physiology. Besides topoisomerases, one of the elements that modulate bacterial DNA supercoiling is the nucleoid-associated protein H-NS [35]. We thus measured the stability of the plasmids in the *E. coli* strain JMG100, an *hns* defective strain derived from *E. coli* MC4100. As shown in Figure 1, the lack of H-NS allowed the maintenance of the plasmid pBS3.25, which harbors Sm*qnr*. This increased stability of the plasmid conferred slightly greater resistance to quinolones than in the wild-type strain (Table 1).

### Mutations Compensating Fitness Costs Associated with the Expression of Sm*qnr* Occur in the Chromosome of *E. coli*

Even though the expression of Sm*qnr* incurs an immediate fitness cost on *E. coli*, this might be alleviated if secondary compensatory mutations are selected for. If these mutations occur in the plasmid containing the gene, its dissemination will be aided. In contrast, if these mutations occur in the chromosome, the plasmid will be lost upon entering a novel host and dissemination precluded. We thus performed an experimental evolution study, making serial passages of *E. coli* KZM120 containing the plasmid pBS3.25 harboring Sm*qnr*. Passages were performed for two replicate isolates 1) in the presence of carbenicillin for selecting the plasmid 2) in the presence of ofloxacin for selecting Sm*qnr*, and 3) alternating the selection pressure (one week carbenicillin, one week ofloxacin) for sequentially selecting the plasmid and the resistance gene. The presence of the plasmid was analyzed after four weeks (around 200 generations) of growth. All cultures grown in the presence of ofloxacin lost the pBS3.25 plasmid harboring Sm*qnr*, whereas it was maintained in cultures exposed to carbenicillin (strains MBS228, MBS229) and under alternating selection with carbenicillin and ofloxacin (strains MBS230 and MBS231).

**Figure 2:**
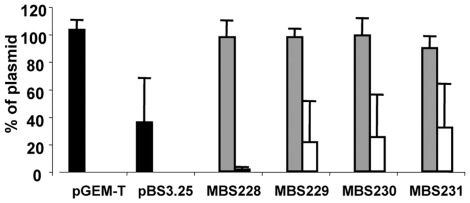
Stability of Sm*qnr*-containing plasmids after experimental evolution. Plasmid stability was estimated in the evolved strains (gray bars) and in *E. coli* KZM120 clones retransformed with plasmids obtained from the evolved strains (white bars). Bars show the percentage of cells containing the plasmids. Black bars pGEM-T, empty vector and MBS25 original strain with pBS3.25 plasmid without evolution; gray bars, evolved strains obtained after growth in the presence of carbenicillin (MBS228 and MBS229) or with carbenicillin and ofloxacin alternatively (MBS230 and MBS231); white bars, strains obtained after transforming *E. coli* KZM120 with the plasmids from the evolved strains MBS228, MBS229, MBS230 and MBS231. As shown, the plasmids were stable in the evolved strains but remained unstable upon their transfer to a new host.

To ascertain whether the mutations stabilizing the plasmids occurred in the plasmids themselves or in the chromosome, the pBS3.25 plasmids obtained from the evolved strains were introduced into the original, non-evolved *E. coli* KZM120 strain. As shown in Figure 2, pBS3.25 plasmids obtained from the evolved strains were lost in a manner similar to that observed for the original pBS3.25 plasmid when introduced into a new host. This suggests that stabilization was not due to mutations in the plasmids. To further confirm this, the plasmids from the evolved strains were extracted and sequenced. No change was detected in the sequence of any of the plasmids; thus, stabilization was only due to chromosomal mutations.

Since Qnr binds bacterial topoisomerases, it may be that compensatory mutations occur in the genes coding for them. We thus sequenced *gyrA*, *gyrB*, *parC* and *parE* in the evolved MBS228, MBS229, MBS230 and MBS231 strains. No mutation was detected in MBS228 or MBS229, while a change at position 87 of GyrA was detected in MBS230 and MBS231. However, this mutation was also found in other strains that lost their plasmids during the ofloxacin evolution experiment (see below); it is responsible for the reduction in quinolone susceptibility shown by these two strains, not for the stabilization of pBS3.25.

**Figure 3:**
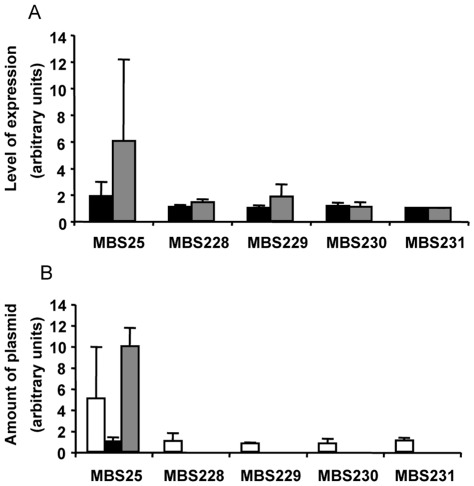
Analysis of the expression of *hns* and Sm*qnr25*, and copy number of the pBS3.25 plasmid upon experimental evolution. Panel A, analysis of expression of *hns* and Sm*qnr*. Black bars, *hns* expression; gray bars, Sm*qnr25* expression. Despite pBS3.25 being stabilized in the *hns* defective mutant, the expression of this gene did not change in the evolved strains, indicating that plasmid stabilization in these isolates is not due to reduced *hns* expression. The copy number of values were normalized taking the value for MBS231 as 1. MBS228, MBS229, MBS230 and MBS231 are the evolved strains and MBS25 the original, non-evolved strain. Panel B. Copy number of pBS3.25 after experimental evolution. White bars, copy number of pBS3.25 plasmid in the evolved strains and in the overall MBS25 population. The large deviation observed in the full MBS25 population reflects that it is formed by two subpopulations, one (small colonies) harboring pBS3.25 in high copy numbers, and another (larger colonies) in which the plasmid copy number is low. The copy number of pBS3.25 in the small colonies is shown with a gray bar; the copy number of pBS2.35 in the colonies of normal size is shown with a black bar. As shown, bacteria evolved to reduce the plasmid copy number and hence express low levels of Sm*qnr*. This reduction is evident even in the original population, which has small colonies with plasmids in high copy number and normal size colonies with low copy numbers of pBS3.25. Values were normalized using the values for the normal-sized colonies of MBS25 (black bar) as 1.

Given the pBS3.25 plasmid stabilization observed in the *hns* defective mutant, *hns* is a potential candidate for acquiring compensatory mutations. The *hns* gene was sequenced in MBS228, MBS229, MBS230 and MBS231. No mutations were detected indicating that compensation is not due to the modification of H-NS. It is possible, however, that even if *hns* is not mutated, its expression might be impaired. We thus compared the level of expression of *hns* in the evolved MBS228, MBS229, MBS230 and MBS231 strains to that in the non-evolved MBS25. No differences were seen (Figure 3), indicating that stabilization is due neither to mutations in *hns* nor to differences in its expression.

An intriguing aspect of strains MBS228 and MBS229 that evolved under selective pressure from carbenicillin is that their susceptibility to quinolones was higher than that seen in the original non-evolved strain, whereas strains MBS230 and MBS231, which evolved under alternating pressure from ofloxacin and carbenicillin, were less susceptible to quinolones than the non-evolved strains (Table S1). Since the level of expression of a given resistance determinant affects the conferred fitness costs [27], and since the level of Sm*qnr* expression correlates with susceptibility to quinolones [28], we measured the expression of Sm*qnr* in the MBS228, MBS229, MBS230 and MBS231 evolved strains and in the non-evolved strain MBS25. The finding that Sm*qnr* expression was lower in the evolved strains indicates that the alleviation of the fitness costs is likely due to the reduced expression of Sm*qnr* (Figure 3). Low Sm*qnr* expression was also observed in the strains MBS230 and MBS231, despite their reduced susceptibility to quinolones, suggesting that, even when selective pressure with quinolones is exerted, *E. coli* evolves towards the acquisition of resistance by chromosomal mutations (see below) and tends to lose the pBS3.25 plasmid containing the Sm*qnr* gene. The benefits of harboring this gene are probably smaller than the burden it generates, even in the presence of the selective antibiotic.

The reduced expression of Sm*qnr* in the evolved strains might be due either to mutations altering this, or to mutations that reduce the copy number of pBS3.25. To determine which was at work, we quantified the amount of plasmid DNA as described in Methods. Figure 3 shows that the amount of pBS3.25 in the evolved clones was smaller than in the original strain, indicating that compensatory mutation(s) reduce the plasmid copy number. Consequently, the expression of Sm*qnr* and the associated fitness costs were lower. This reduction in plasmid copy numbers and its effect on *E. coli* fitness was evident from the beginning of the experimental evolution experiment. Indeed, we detected that even the non-evolved strain showed diversity in terms of the colony size, with some of the population forming regular colonies and some forming small colonies. We thus measured the amount of pBS3.25 in both types. As shown in Figure 3, the small colonies had higher copy numbers of pBS3.25 than the larger ones. This indicates the co-existence of fitter derivatives with low copy numbers of pBS3.25 and low expression of Sm*qnr* that overcome the less fit derivatives, which express higher levels of Sm*qnr*.

### Experimental Evolution in the Presence of Quinolones Selects for Chromosomal Mutations Allowing Plasmid Loss

An intriguing aspect of the evolved strains MBS230 and MBS231 is their showing lower susceptibility to quinolones than the non-evolved MBS25 (Table S1), despite the former having lower copy numbers of pBS3.25 than the latter. This suggests that they acquired chromosomal mutation(s) leading to this reduced susceptibility. Since the main mechanism of quinolone resistance consists of mutations in the quinolone resistance determinant regions (QRDRs) of the genes *gyrA*/*B* and *parC*/*E*, which code for bacterial topoisomerases, the QRDRs of *gyrA*, *gyrB*, *parC* and *parE* were sequenced. An amino acid change in GyrA - D87Y - was found in both MBS230 and MBS231.

**Figure 4:**
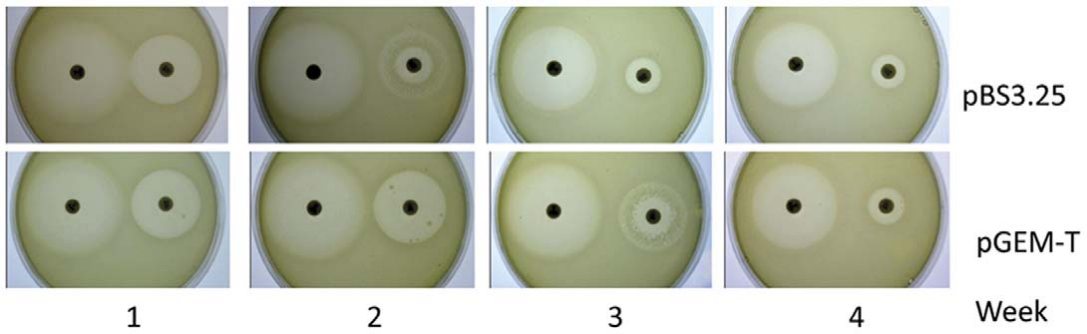
Selection of chromosomally-encoded quinolone resistance in strains containing - or not containing - Sm*qnr*. The figure shows the zones of inhibition of two evolved clones (one containing pBS3.25, the other the pGEM-T vector) over four weeks of evolution under selective pressure with ofloxacin. Left disk, ofloxacin (5 µg), right disk, nalidixic acid (30 µg). As shown, some scattered resistant colonies appeared inside the nalidixic acid inhibition zone that, after evolution displaced the otherwise susceptible population. The selection of resistance was independent of the presence of the SmQnr-coding plasmid pBS3.25.

Low-level resistance to antibiotics can allow bacteria to develop high-level resistance more easily because it allows persistence of sufficient number of cells in the presence of the selector, facilitating the random emergence of mutants. Thus, to ascertain whether or not the presence of the pBS3.25 plasmid containing the Sm*qnr* gene increases the capability of *E.coli* to acquire quinolone resistance, new sets of experimental evolution experiments were performed. Independent cultures of *E. coli* containing either pBS3.25 or pGEM-T were subjected to serial passages in the presence of ofloxacin (8, 16 or 32 ng/ml) for four weeks. In all cases, the evolved clones showed lower susceptibility to quinolones irrespective of their harboring the Sm*qnr* gene. Further, inspection of the inhibition zones around the quinolone-containing disks revealed the presence of resistant colonies one week after quinolone challenge in some of the evolving clones, irrespective of whether the plasmid harbored Sm*qnr* (Figure 4). These results show the existence of a mixed population of resistant and susceptible bacteria in the culture. Inspection of the quinolone inhibition zones at weeks 2, 3 and 4 showed that the resistant population displaced the susceptible one over time (Figure 4), a feature that fits with the recent description of selection of quinolone resistance at sub-inhibitory concentrations of antibiotics as the consequence of differential fitness in the presence or in the absence of antimicrobials [36]. The QRDR regions of genes *gyrA*/*B* and *parC*/*E* from the evolved clones were sequenced. Most evolved clones (90%) showed a change at position 87 of GyrA (Table S1), as seen in MBS230 and MBS231, which evolved under alternating selective pressure from carbenicillin and ofloxacin. Since this mutation is present in the strains containing pGEM-T (Table S1), and since all the clones containing pBS3.25 evolving in the presence of ofloxacin alone lost this plasmid, this mutation was clearly not selected to compensate for the fitness costs associated with Sm*qnr* in MBS230 and MBS231 (which kept the plasmid pBS3.25). Among the other evolved clones, MBS237 showed an S83L amino acid change in GyrA, MBS232 showed a G466A change in GyrB, and MBS258 showed the deletion of two amino acids (L,G), at positions 474 and 475 of GyrB. Two clones (MBS244 and MBS253) showed no mutation within the QRDRs of *gyrA*/*B* and *parC*/*E*; their phenotypes might be due to changes in the level of expression of other genes such as those coding for porins or for multidrug efflux pumps.

## Discussion

Environmental bacteria together possess a huge number of resistance determinants [22] that could confer resistance to antibiotics upon their transfer to a heterologous host (resistome). However, the number of resistance genes actually acquired by human pathogens though HGT is very low, and when it has occurred the original environmental microbial host has usually remained unknown [6], [19], [37]. There are four main bottlenecks that might account for this. The first is ecological connectivity, which is a needed condition. If the potential donor of resistance never shares its habitat with pathogenic bacteria, the resistance gene has no chance of being transferred. Secondly, even if they share the same habitat, the potential donor and the recipient may not belong to the same genetic exchange community, understood as the cohort of bacterial species among which DNA transfer can occur [38]. Thirdly, a founder effect may exist, understood as the ‘first gene to come being the one to win’. This implies a large degree of stochasticity in the acquisition and spread of resistance. If a resistance gene is incorporated into a proficient mobile genetic element, and is acquired by a competitive bacterial clone, it is likely that it will rapidly spread under antibiotic selective pressure. This can occur locally, in which case different genes can arise at different places or, if the elements/clones that allow its dissemination are highly epidemic, one single gene can be disseminated. Once this gene is disseminated, the chances of another gene conferring the same type of resistance entering and spreading among the population would be low; once bacteria are resistant, there is no further selective pressure for replacing one gene by another. The last bottleneck is the fitness cost. If the acquisition of a given resistance determinant incurs a high fitness cost, its chances of remaining in the bacterial population will be low; the resistant bacteria will be outcompeted by their susceptible counterparts in the absence of selection [39]. In this regards, it has been discussed that the probability of a successful HGT event is strongly affected by the number of interactions that a protein can make with its neighbors [40], [41]. These bottlenecks are not mutually exclusive, and combinations might be at work in specific evolution processes.

To analyze the importance of these mechanisms in the evolution of antibiotic resistance, we determined the stability of plasmids encoding either the widely-spread *qnrA* gene or the *S. maltophilia* Sm*qnr* gene, both of which code for low-level quinolone resistance. *S. algae*, the original host of *qnrA*, is a water-dwelling bacterium rarely involved in infections; there is therefore probably only a small chance of quinolone-resistance being transferred to *E. coli*. In contrast, *S. maltophilia* is an opportunistic pathogen and thus the chance of Sm*qnr* being transferred to a human pathogen under selective antibiotic pressure is higher. Even so, the literature contains no reports of the transfer of Sm*qnr* to another bacterium, suggesting that ecological connectivity is not the major bottleneck affecting the prevalence of a given *qnr* gene in bacterial pathogens. The fact that the plasmid encoding the QnrA protein was stable in the studies made in the present work, whereas the plasmid encoding SmQnr was rapidly lost by *E. coli*, shows that differential allelic fitness costs likely explain much of the probability of quinolone resistance genes being spread among bacterial populations.

**Figure 5:**
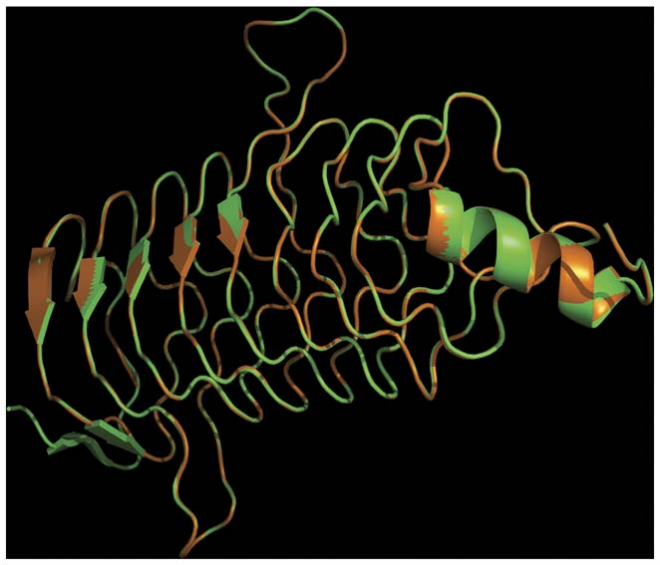
Overlapping of QnrA1 and SmQnr tertiary structures. The structures of SmQnr and QnrA1 were modeled at http://swissmodel.expasy.org/in the automated mode, using the structure of the QnrB1 protein as a template [61]. Both predicted structures were overlapped using MacPyMOL. As shown in the Figure, the predicted structures were very similar. The program did not allow the first 13 amino acids of SmQnr and the first 4 amino acids of QnrA1 to be modeled. Green QnrA1, orange SmQnr.

The experimental evolution experiments performed permitted the selection of compensatory mutations that allowed plasmid maintenance. However, these mutations occurred in the bacterial chromosome, and reduced the plasmid copy number. The plasmid used in these experiments harbours a ColE1 origin of replication. The regulation of copy number of this family of plasmids involves several chromosomal genes, some of which deal with plasmid replication, some with dimers resolution and even some others as that encoding the bacterial tryptophanase, which are involved in the basic bacterial metabolism [42], [43], [44], [45]. The identity of the compensatory mutations remains to be established.

A decrease in plasmid copy number reduces the resistance to quinolones. Thus, in the presence of antibiotics, the evolved mutants lost the gain in fitness conferred by the plasmid encoding SmQnr (lower susceptibility to quinolones) without higher transmissibility of Sm*qnr*; the plasmid remained unstable upon transferring to a new host. Even when quinolones were present, the SmQnr-containing plasmid was lost, and the quinolone-resistant mutants mainly showed mutations in the genes coding for bacterial topoisomerases. Further, the co-existence of susceptible and resistant populations in the same culture, and the finding that resistant mutants overcome the susceptible population over time, indicates that concentrations of antibiotic that do not fully inhibit bacterial growth can select for antibiotic resistance [36].

It is generally accepted that fitness costs due to the acquisition of a plasmid-encoded antibiotic resistance gene mainly derive from the metabolic load involved in the replication, transcription and translation of the mobile element. The present results show, however, that fitness costs can be much more specific than expected, and derive from the epigenetic compatibility of the determinants encoded by the plasmid with those encoded by the host genome. Recently published results indicating that epistatic effects are critical in the acquisition of multidrug resistance [46] and in the evolution of bacterial populations [47] are in line with the present findings.

Although the similarity of the primary structures of QnrA and SmQnr is low, they belong to the pentapeptide repeats family, and model predictions suggest a remarkable similarity in their tertiary structure with just a few differences at the N terminus (Figure 5). The present results therefore indicate that, even for antibiotic resistance determinants with a very similar structure and mechanism of action, fitness costs can vary greatly, and that these are due to specific changes in bacterial physiology more than to any general, non-specific, metabolic burden.

The finding that SmQnr expression incurs no large fitness cost in an *hns* mutant strongly supports the idea that SmQnr disturbs *E. coli* regulatory networks. H-NS is a nucleoid-associated protein that modulates the expression of several genes in the latter species [48] and can silence the expression of HGT-acquired genes in *Enterobacteriaceae* [49]. The effect we observed cannot be attributed to silencing since the susceptibility to quinolones conferred by the plasmid encoding SmQnr was slightly lower in the *hns* mutant than that in the wild-type strain. Further, silencing should allow stabilization in the wild-type strain and impair plasmid maintenance in the *hns* mutant, the opposite to that actually observed. Since H-NS can modulate DNA supercoiling [35], and since Qnr proteins bind bacterial DNA topoisomerases [50], [51], it might be that the effect of SmQnr on *E. coli* physiology derives from a stronger effect of this protein than that of QnrA1 on DNA supercoiling. Alternatively, the effect might be due to a specific effect on some of the genes that are regulated by H-NS. In this regard, it has recently been proposed that the connectivity of an HGT-acquired protein with the cellular networks of the new host, rather than its function, provides a barrier to HGT [40].

Antibiotic resistance genes have been said to originate in producer organisms in which they play an autoprotection role, similar to the function they have after their transfer to human pathogens [1], [2], [52]. However, although the resistance genes acquired by human pathogens code for proteins in the same families as found in antibiotic producers [1], there are still few examples in which a direct origin can be unequivocally tracked. One of these cases involves the *qnrA* gene; this has been found in the chromosome of different strains of *S. algae* without any apparent evidence of its recent acquisition [14]. Since *S. algae* is not an antibiotic producer, and since quinolones are synthetic antibiotics, it is reasonable to think that Qnr determinants cannot have been selected for avoiding the activity of quinolones. Indeed, it has been suggested that Qnr proteins are involved in the adaptation of bacterial cells to different stresses, including naturally occurring DNA-damaging agents [53] and cold-shock [54].

A central dogma in evolution is that a change in the function of a given protein occurs as the consequence of mutations that are selected under a particular type of pressure. It is thus tempting to speculate that those determinants whose original function is not antibiotic resistance are pre-resistance genes that evolved towards resistance by the selection of proficient mutant variants in the presence of antibiotics. Since the QnrA1 determinant present in human pathogens varies by 3 or 4 amino acids to those currently described for *S. algae*, it may be that this gene evolved towards resistance from a former pre-resistance gene. However, the fact that the Qnr elements encoded in the chromosomes of *S. algae* confer low-level quinolone resistance to *E. coli* goes against this hypothesis. The term exaptation was coined to describe an evolutionary process in which a given element acquires a new function, not because the element itself changes, but because the environment changes [21]. Our data indicate that, at least for Qnr determinants, exaptation might be an important evolutionary process in the acquisition of resistance to antibiotics by human bacterial pathogens.

## Materials and Methods

### Bacterial Strains, Plasmids and Growth Conditions

The bacterial strains and plasmids used in this work are described in Table 2. All strains were grown in Luria-Bertani (LB) broth [55] at 37°C.

**Table 2:** Strains and plasmids used in this work.

Strain	Description	Reference or source
*Escherichia coli* KZM120	*ΔacrAB*::Tn903 Kan^r^	[34]
*Escherichia coli* MC4100	*araD139 Δ(argF-lac)U196 rpsL150 relA1 deoC1 ptsF25 rbsR flb B5301*	[59]
*Escherichia coli* JMG100	MC4100 *hns*-90::Tn10	[60]
*Shewanella algae* KB-1	Strain with the gene *qnrA3* in its chromosome.	[14]
*Shewanella algae* KB-2	Strain with the gene *qnrA4* in its chromosome.	[14]
**Plasmid**		
pGEM-T	Cloning vector with polyA, *amp^r^*	Promega
pBS3.25	pGEM-T with Sm*qnr* of *S. maltophilia* E847	[15]
pBS18	pGEM-T with *qnrA1* of plasmid pMG252 of *Klebsiella pneumoniae*	This work
pBS19	pGEM-T with *qnrA3* of *Shewanella algae* KB-1	This work
pBS20	pGEM-T with *qnrA4* of *Shewanella algae* KB-2	This work
pMG252	Clinical plasmid with the gene *qnrA1*	[10]

### DNA Manipulation, PCR and Sequencing

The genes *qnrA1*, *qnrA3* and *qnrA4* were obtained by PCR, using the Expand Long Template PCR System (Roche) and the primers QnrAfor and QnrArev (Table S2), using as templates the plasmid pMG252 for *qnrA1*, and chromosomal DNA from *Shewanella algae* KB-1 and KB-2 for *qnrA3* and *qnrA4* respectively. The reactions had one denaturation step at 94°C for 2 min, followed by 10 amplification cycles: 94°C for 30 s, 50°C for 30 s, and 68°C for 2 min, followed by 20 further amplification cycles: 94°C for 30 s, 55°C for 30 s, and 68°C for 2 min, with a final extension step of 68°C for 7 min. The PCR products were electrophoresed in 1% agarose gels with TAE, removed from the gel using the GFX™ PCR DNA and Gel Band Purification Kit (GE Healthcare), and cloned into the pGEM-T vector (Promega), generating the plasmids pBS18, pBS19 and pBS20, which contained the *qnrA1*, *qnrA3* and *qnrA4* genes respectively. These were used to transform *E. coli* KZM120, MC4100 and JMG100 strains as previously described [56]. The *qnr*-cloned genes were sequenced by Macrogen (http://dna.macrogen.com/eng/) to assure that no-mutation was introduced during the cloning procedure.

The QRDRs of *gyrA*, *gyrB*, *parC* and *parE* were amplified using the PCR Master Mix (Promega) and previously described primers [57]. The complete topoisomerase genes and the *hns* gene were amplified using the primers gyrA1/gyrA8, gyrB1/gyrB8, parC1/parC6, parE1/parE6 and Hns1/Hns2 respectively (Table S2). PCR products were purified using the QIAquick PCR Purification Kit (QIAGEN) and sequenced at Macrogen (http://dna.macrogen.com/eng/) using the primers described in Table S2.

### Real Time PCR and RT-PCR

To analyze the expression of *hns* and Sm*qnr25*, the strains were grown overnight in LB broth with 100 µg/ml carbenicillin at 37°C. The cultures were then diluted to O.D._600_ = 0.01 and grown under the same conditions until the exponential phase (O.D._600_ = 0.3–0.4). Total RNA was isolated from 30 ml of exponential cultures using the RNeasy Mini Kit (QIAGEN, Valencia, CA), treated with Turbo DNA-free reagent (Ambion, Inc., Austin, TX) and quantified in a Nanodrop spectrophotometer. 400 ng of each sample were used for reverse transcription with the High-Capacity cDNA Reverse Transcription Kit (Applied Biosystems). Real-time PCR was performed in a 7300 Real-Time PCR System (Applied Biosystems, Foster, CA, USA), using SYBR Green PCR Master Mix (Applied Biosystems) and the primers RThnsfwd/rv for the *hns* gene and RTqnr25fwd/rv for Sm*qnr25* gene (Table S2). RNA samples corresponding to four independent experiments were analyzed. The *gapA* gene (primers RTgapAfwd/rv Table S2) was used to normalize the data. The relative amount of mRNA for *hns*, and Sm*qnr25* versus the internal controls (*gapA*), was calculated following the 2^–ΔΔCt^ method [58]. The absence of genomic DNA contamination was verified by real-time PCR of the RNA samples before reverse transcription.

The conditions described above were used to determine the pBS3.25 plasmid copy number. For this, the copy number of the *bla*_TEM1_ gene, present in the plasmid backbone and conferring resistance to ß-lactams, was determined using the primers ampRF/R (Table S2) by real-time PCR as described above. The plasmid copy number was calculated using a standard curve made using known pBS3.25 copy numbers in the real-time PCR reaction. All data were normalized to that obtained for the colonies showing a normal size, which received a value 1.

### Plasmid Stability

To estimate the number of cells in a given population containing the plasmid, sequential dilutions of the cultures to be tested were seeded onto LB plates containing either 100 µg/ml carbenicillin, or no antibiotic, and the number of colony forming units (c.f.u.) recorded after culturing the plates for 24 h at 37°C. The number of cells carrying the plasmid was estimated as the ratio of c.f.u. in the presence of carbenicillin and total c.f.u. (100%). Data for each plasmid are the results of at least four independent experiments.

### Susceptibility to Antibiotics

The susceptibility of *E. coli* MC4100, JMG100 and their derivatives was analyzed in Mueller Hinton agar (Pronadisa) containing 0.5 mM isopropyl-thio-ß-D-galactopyranoside (IPTG) using the Epsilon test (AB Biodisk) following the manufacturer’s instructions. The susceptibility of the evolved *E. coli* KZM120 (pBS3.25) and *E. coli* KZM120 (pGEM-T) derived clones was analyzed by the agar dilution method in Mueller Hinton agar containing 0.5 mM IPTG. Each assay was performed at least four times; results were recorded after 24 h of incubation at 37°C.

### Experimental Evolution Assays

*E. coli* KZM120 (pBS3.25) was grown in LB containing each of the following antibiotics: 100 µg/ml carbenicillin; 8 ng/ml ofloxacin (1/2 CMI); 16 ng/ml ofloxacin (CMI); 32 ng/ml ofloxacin (2X CMI). All cultures were grown for four weeks, with daily 1∶1000 dilutions of fresh medium and the corresponding antibiotic. The same procedure was followed using alternate selection pressures, i.e., one week with 8 ng/ml ofloxacin and then with 100 µg/ml carbenicillin. As a control for the selection of quinolone resistant mutants in the absence of Sm*qnr*, the same experimental evolution assays were preformed with *E. coli* KZM120 (pGEM-T) challenged with 8 ng/ml ofloxacin (CMI), 16 ng/ml ofloxacin (2X CMI) and 32 ng/ml ofloxacin (4X CMI).

## Supporting Information

Table S1 Quinolone susceptibility and mutations in their QRDR of in vitro evolved clones of E. coli KZM120 pBS3.25 (MBS228-MBS251) and pGEM-T (MBS252-MBS270).
(DOCX)

Table S2 Primers used in this work.
(DOCX)
