# Supplementary material for: Differential Epigenetic Compatibility of qnr Antibiotic Resistance Determinants with the Chromosome of Escherichia coli
Source: PLoS One. 2012 May 4;7(5):e35149. doi: 10.1371/journal.pone.0035149 (PMC3344834; doi:10.1371/journal.pone.0035149)
Supplement: Table S1 — Quinolone susceptibility and mutations in their QRDR of in vitro evolved clones of E. coli KZM120 pBS3.25 (MBS228-MBS251) and pGEM-T (MBS252-MBS270). (DOCX) [file pone.0035149.s001.docx]

**Table S1. Quinolone susceptibility and mutations in their QRDR of *in vitro* evolved clones of *E. coli* KZM120 pBS3.25 (MBS228-MBS251) and pGEM-T (MBS252-MBS270).**

| **Plasmid** | **Selective pressure*** | **Strain** | **Mutation in QRDR** | | | | **MICs (µg/ml)** | | | |
| --- | --- | --- | --- | --- | --- | --- | --- | --- | --- | --- |
|  |  |  | **GyrA** | **GyrB** | **ParC** | **ParE** | **MOX** | **CIP** | **NAL** | **OFX** |
| None | Non evolved | KZM120 | -**^a^** | - | - | - | 0.008 | 0.004 | 1 | 0.008 |
| pBS3.25 | Non evolved | MBS25 | ND | ND | ND | ND | 0.016 | 0.008 | 1 | 0.016 |
|  | CAR | MBS228^b^ | - | - | - | - | 0,004 | 0.004 | 1 | 0.008 |
|  | CAR | MBS229^b^ | - | - | - | - | 0,004 | 0.004 | 1 | 0.008 |
|  | OFX8/CAR | MBS230^b^ | Asp87Tyr | - | - | - | 0,032 | 0.032 | 32 | 0.064 |
|  | OFX8/CAR | MBS231^b^ | Asp87Tyr | - | - | - | 0,032 | 0.032 | 32 | 0.064 |
|  | OFX8 | MBS232 | - | Glu466Ala | - | - | 0,032 | 0.008 | 2 | 0.032 |
|  | OFX8 | MBS233 | Asp87Gly | - | - | - | 0,064 | 0.032 | 32 | 0.064 |
|  | OFX8 | MBS234 | Asp87Gly | - | - | - | 0,064 | 0.032 | 32 | 0.064 |
|  | OFX8 | MBS235 | Asp87Asn | - | - | - | 0,064 | 0.032 | 32 | 0.128 |
|  | OFX8 | MBS236 | Asp87Gly | - | - | - | 0,064 | 0.032 | 32 | 0.064 |
|  | OFX8 | MBS237 | Ser83Leu | - | - | - | 0,128 | 0.064 | 64 | 0.256 |
|  | OFX8 | MBS238 | Asp87Asn | - | - | - | 0,064 | 0.032 | 32 | 0.064-0.128 |
|  | OFX8 | MBS239 | Asp87Asn | - | - | - | 0,032 | 0.032 | 32 | 0.064 |
|  | OFX8 | MBS240 | Asp87Tyr | - | - | - | 0,064 | 0.032 | 32 | 0.064 |
|  | OFX8 | MBS241 | Asp87Tyr | - | - | - | 0,032-0,064 | 0.032 | 32 | 0.064 |
|  | OFX8 | MBS242 | Asp87Tyr | - | - | - | 0,128 | 0.032 | 32 | 0.064 |
|  | OFX8 | MBS243 | Asp87Asn | - | - | - | 0,128 | 0.032 | 32 | 0.128 |
|  | OFX16 | MBS244 | - | - | - | - | 0,128 | 0.016 | 16 | 0.128 |
|  | OFX16 | MBS245 | Asp87Asn | - | - | - | 0,064 | 0.032 | 32 | 0.128 |
|  | OFX16 | MBS246 | Asp87Tyr | - | - | - | 0,064 | 0.032 | 32 | 0.064 |
|  | OFX16 | MBS247 | Asp87Tyr | - | - | - | 0,064 | 0.032 | 32 | 0.128 |
|  | OFX32 | MBS248 | Asp87Tyr | - | - | - | 0,064 | 0.032 | 32 | 0.064-0.128 |
|  | OFX32 | MBS249 | Asp87Tyr | - | - | - | 0,064 | 0.032 | 32 | 0.064-0.128 |
|  | OFX32 | MBS250 | Asp87Asn | - | - | - | 0,064 | 0.032 | 32 | 0.064-0.128 |
|  | OFX32 | MBS251 | Asp87Asn | - | - | - | 0,064 | 0.032 | 32 | 0.064-0.128 |
| pGEM-T | OFX8 | MBS252 | Asp87Ala | - | - | - | 0,032-0,064 | 0.032 | 32 | 0.064 |
|  | OFX8 | MBS253 | - | - | - | - | 0,008 | 0.004 | 1 | 0.016 |
|  | OFX8 | MBS254 | Asp87Tyr | - | - | - | 0,064 | 0.032 | 32 | 0.064 |
|  | OFX8 | MBS255 | Asp87Tyr | - | - | - | 0,064 | 0.032 | 32 | 0.064 |
|  | OFX8 | MBS256 | Asp87Asn | - | - | - | 0,064 | 0.032 | 32 | 0.128 |
|  | OFX8 | MBS257 | Asp87Asn | - | - | - | 0,064 | 0.032 | 32 | 0.128 |
|  | OFX8 | MBS258 | - | ∆LeuGly474/475 | - | - | 0,016 | 0.016 | 2 | 0.032 |
|  | OFX8 | MBS259 | Asp87Asn | - | - | - | 0,128 | 0.032 | 32 | 0.128 |
|  | OFX8 | MBS260 | Asp87Asn | - | - | - | 0,064 | 0.032 | 32 | 0.128 |
|  | OFX8 | MBS261 | Asp87Asn | - | - | - | 0,064 | 0.032 | 32 | 0.128 |
|  | OFX8 | MBS262 | Asp87Gly | - | - | - | 0,064 | 0.032 | 32 | 0.064 |
|  | OFX16 | MBS263 | Asp87Tyr | - | - | - | 0,064 | 0.032 | 32 | 0.064 |
|  | OFX16 | MBS264 | Asp87Asn | - | - | - | 0,064 | 0.032 | 32 | 0.064 |
|  | OFX16 | MBS265 | Asp87Tyr | - | - | - | 0,064 | 0.032 | 32 | 0.064 |
|  | OFX16 | MBS266 | Asp87Tyr | - | - | - | 0,064 | 0.032 | 32 | 0.064 |
|  | OFX32 | MBS267 | Asp87Tyr | - | - | - | 0,064 | 0.032 | 32 | 0.064 |
|  | OFX32 | MBS268 | Asp87Tyr | - | - | - | >0,128 | >0.128 | >64 | 0.512 |
|  | OFX32 | MBS269 | Asp87Tyr | - | - | - | 0,064 | 0.032 | 32 | 0.064 |
|  | OFX32 | MBS270 | Asp87Gly | - | - | - | >0,128 | >0.128 | >64 | 0.512 |
| pBS18 | Non evolved | MBS198 | ND | ND | ND | ND | 0.128 | 0.064 | 4 | 0.256 |
| pBS19 | Non evolved | MBS211 | ND | ND | ND | ND | 0.064 | 0.064 | 4 | 0.128 |
| pBS20 | Non evolved | MBS212 | ND | ND | ND | ND | 0.064 | 0.064 | 4 | 0.128 |

^a^- No mutation. ^b^ Complete topoisomerase genes sequenced. ND: Not done

*Selective pressure: CAR: 100 µg/ml carbenicillin; OFX8/CAR: 8 ng/ml ofloxacin/ 100µg/ml carbenicillin; OFX8: 8 ng/ml ofloxacin; OFX16: 16 ng/ml ofloxacin; OFX32: 32 ng/ml ofloxacin.
