# Supplementary material for: Differential Epigenetic Compatibility of qnr Antibiotic Resistance Determinants with the Chromosome of Escherichia coli
Source: PLoS One. 2012 May 4;7(5):e35149. doi: 10.1371/journal.pone.0035149 (PMC3344834; doi:10.1371/journal.pone.0035149)
Supplement: Table S2 — Primers used in this work. (DOCX) [file pone.0035149.s002.docx]

**Table S2. Primers used in this work.**

| **Primer** | **Sequence 5´to 3´** |
| --- | --- |
| QnrAfor | AGCCGTATGGATATTATTG |
| QnrArev | CCAGAGCTAATCCGGCAGC |
| Hns1 | ATGAGCGAAGCACTTAAAATTC |
| Hns2 | TTATTGCTTGATCAGGAAATCG |
| gyrA1 | ATGAGCGACCTTGCGAGAG |
| gyrA2 | ATACGGAATTTCGTGGACG |
| gyrA3 | CGTACCGGTCGCGGCAAGG |
| gyrA4 | TTTTTCGTGCTCAAGACCG |
| gyrA5 | GGAGCCAGAGTTCGGCGTG |
| gyrA6 | GTTTGATCGCCACTTTACC |
| gyrA7 | GATCCTGCCAGTGACCGAG |
| gyrA8 | TTATTCTTCTTCTGGCTCG |
| gyrB1 | ATGTCGAATTCTTATGACTC |
| gyrB2 | AATACCGTCTTTTTCAGTGG |
| gyrB3 | CTATGAAGGCGGCATCAAGG |
| gyrB4 | ATTTTACCCTTCAGCGGCAG |
| gyrB5 | TTCCGAACTGTACCTGGTGG |
| gyrB6 | ATCAAACTTCCACTGGCTGC |
| gyrB7 | CGTTATCCGAAAGCAATGC |
| gyrB8 | TTAAATATCGATATTCGCCG |
| parC1 | ATGAGCGATATGGCAGAGCG |
| parC2 | AGTACGCGCGCACCTGAAAC |
| parC3 | TTCAGTGCGTATGCGCGCGG |
| parC4 | CACAATGGTGACAGGTTCAG |
| parC5 | TGCAGGAACGCGAAGAAGCG |
| parC6 | TTACTCTTCGCTATCACCGC |
| parE1 | ATGACGCAAACTTATAACGC |
| parE2 | TCCACAGCTTCAGTATCACC |
| parE3 | CTTTTAAAGATGAGATCAAC |
| parE4 | TCTTACCTTTCAGTGGCATG |
| parE5 | CTGGCTGATTGTACCGCGC |
| parE6 | TTAAACCTCAATCTCCGCC |
| RThnsfwd | GCTGATCGCTGACGGTATTGA |
| RThnsrv | ATTTAACGGCAGCAAGGCTATT |
| RTgapAfwd | TGTTTTCCGTGCTGCTCAGA |
| RTgapArv | CGTCTAACAGGTCGTTGATTGC |
| RTqnr25fwd | AAGTCGAACCTGCGCTATGC |
| RTqnr25rv | CCACAGCTCGCACTTTTCC |
| ampRF | CGCCCCGAAGAACGTTT |
| ampRR | CGCGCCACATAGCAGAACTT |
